# Transitions for older people with intellectual disabilities and behaviours that challenge others: A rapid scoping review

**DOI:** 10.1111/jar.13054

**Published:** 2022-11-26

**Authors:** Elizabeth Tilley, Joanne Jordan, Mary Larkin, Jitka Vseteckova, Sara Ryan, Louise Wallace

**Affiliations:** ^1^ Faculty of Wellbeing, Education and Language Studies The Open University Milton Keynes UK; ^2^ Faculty of Health, Psychology and Social Care Manchester Metropolitan University Manchester UK

**Keywords:** ageing, behaviours that challenge others, intellectual disabilities, older people, transitions

## Abstract

**Background:**

People with intellectual disabilities and behaviours that challenge others are living longer. This review aimed to explore what is known about the health and social care needs, experiences, service interventions and resources of and for this population as they transition to different care contexts in the UK.

**Method:**

A rapid scoping review of published and unpublished literature was conducted based on collaborative working with key stakeholders and using systematic methods of data searching, extraction and analysis.

**Results:**

Consistent social work support, skilled staff, suitable accommodation, creative engagement with individuals and families to plan ahead, and timely access to quality healthcare are all required to promote successful transitions as people age, and to avoid unwanted/inappropriate transitions at points of crisis.

**Conclusions:**

More research is needed to assess the types of services that this population can and do access as they age, the quality of those services, and the extent to which local commissioners are planning ahead for people with intellectual disabilities and behaviours that challenge others.

## 1 INTRODUCTION

### 1.1 Background and rationale

Over 1 million adults with intellectual disabilities live in the United Kingdom (UK), representing over 2% of the total adult population (Mencap, 2021). In line with an overall UK ageing population (Office of National Statistics (ONS), 2021) people with intellectual disabilities are living longer. In England, an estimated 53% of the total population of people with intellectual disabilities were aged 45+ in 2020 (with almost 5% aged 85+), predicted to rise to 56% by 2040 (and 7.5% aged 85+) (Institute of Public Care (IPC), 2020). Increased longevity is heavily implicated in the predicted rise of 30% in people with intellectual disabilities aged 50+ requiring social care services in England over the period 2012–2030 (Emerson et al., 2012). In Wales, in 2020/21, 9% of all people with intellectual disabilities registered (1231 people) were aged 65+, an increase of 54% from 2001/02, with 18% of people aged 65+ (222 people) living with their parents or other family members (StatsWales, 2022). Data from the 2011 census showed that 37% of people with intellectual disabilities in Scotland were aged 45+ (9619 people), with 3% aged 75+ (Scottish Learning Disabilities Observatory, n.d.). The 2011 census also reported that 24% of people in Northern Ireland with intellectual disabilities were aged 45+ (9049 people), with 8% aged 65+ (Learning Disability Data and Northern Ireland Consortium, 2020).

Although the means by which the above estimates are calculated vary, using different ways to count people and at different points in time, the numbers highlight that the needs of older people with intellectual disabilities across the UK four nations (England, Wales, Scotland and Northern Ireland) require attention from policy‐makers, commissioners and service providers. In addition, they are likely to underestimate the true levels of need, as many people with intellectual disabilities are not known to health or social services (Public Health England (PHE), 2016a; National Institute for Health and Care Excellence (NICE), 2018a) and the known proportion falls as people age (Emerson et al., 2012).

UK based studies have for some years found that older people with intellectual disabilities experience more health‐related problems than the general population, with these issues often occurring at a younger age (De Winter et al., 2014; De Winter et al., 2016; Robertson et al., 2015; Strydom et al., 2013), including onset of conditions such as dementia (Ballard et al., 2016). A growing body of international evidence, such as the large‐scale longitudinal studies IDS‐TILDA in Ireland, the Healthy Ageing and Intellectual Disabilities Study in the Netherlands, and SAge‐ID in Australia (de Leeuw et al., 2022; McGlinchey et al., 2019; Reppermund & Trollor, 2016) mirrors the UK experience, giving rise to discussions around ‘premature ageing’ (Haveman et al., 2011; Schoufor et al., 2013).

Older people with intellectual disabilities also face additional challenges associated with their circumstances of care and support. In the UK these include: family carers (typically parents) becoming unable to continue to provide care (Cairns et al., 2013; Carers Trust, 2020; Pryce et al., 2017); lack of future planning (Brennan et al., 2020; Lee & Burke, 2020; Walker & Hutchinson, 2018); and, limitations in the availability of appropriate community residential accommodation (NICE, 2018a; Taggart & Hanna‐Trainor, 2017; Turner & Bernard, 2014). Similar challenges have been identified in other national contexts, as social care staff working in supported accommodation are tasked with providing care to a growing population of older people with intellectual disabilities and complex healthcare needs (Alftberg et al., 2021; Northway et al., 2017). Lack of appropriate accommodation and insufficient numbers of suitably trained staff, particularly in the context of responding to the changing needs of people with intellectual disabilities as they age (e.g., with the onset of age related conditions like dementia; onset of frailty; the requirement for home adaptations and introduction of new equipment like hoists etc.), have been identified as two key factors working against government policies that promote the concept of ‘ageing in place’ (Brennan et al., 2020; Northway et al., 2017).

Some people with intellectual disabilities are known to display behaviours that challenge others. These behaviours may be a form of communication or produce sensory stimulation for the person (The Challenging Behaviour Foundation, 2021). Within the context of this article we use the phrase ‘behaviours that challenge others’ as a means of acknowledging their inherently relational and socially constructed nature (Mansell, 2007). As such, they are a product of: environmental factors (such as: the response of professional carers; the quality of the material environment; how well a service is organised; the quality of commissioning processes) and individual characteristics (such as: the presence of sensory disabilities or mental health issues; the onset of new conditions like dementia; events in a person's life history) (Mansell, 2007; Norfolk Safeguarding Adults Board, 2021). While policy and guidance across the UK since the 1990 s has stressed the socially constructed nature of ‘challenging behaviour’, it is clear that in some contexts the term continues to be used to label people (Mansell, 2007; Royal College of Psychiatrists, 2016). Such use can be driven by a need to serve agendas other than improving a person's care or outcomes, such as to enhance the legitimacy of a service, to justify service practices or to empower management decisions (Haydon‐Laurelut & Nunkoosing, 2016).

Internationally, it is estimated that up to 20% of adults with intellectual disabilities display behaviours that challenge others (Bowring et al., 2019; Bowring et al., 2017; Jones et al., 2008; Lundqvist, 2013). For some, the behaviour is consistently displayed over long periods; for others it is more sporadic, depending upon their personal circumstances and on how well services meet their needs over time (Mansell, 2007). People with intellectual disabilities who present a challenge to others comprise a very diverse group, including those with mild intellectual disabilities who have been diagnosed as mentally ill, those who enter the criminal justice system for crimes such as arson or sexual offences, or those with severe or profound intellectual disabilities who injure themselves (Mansell, 2007; NICE, 2018a). There is limited evidence concerning the epidemiology of behaviours that challenge others amongst older people with intellectual disabilities (Davies & Oliver, 2013). Although some studies indicate a decline in these behaviours with advancing age (Holden & Gitlesen, 2006; Jones et al., 2008; Murphy et al., 2005), others have found their persistence as people get older (Lundqvist, 2013; O'Dwyer et al., 2018; Taylor et al., 2011; Totsika et al., 2008).

Research suggests that behaviours that challenge others may prompt the need for older people with intellectual disabilities to move to new homes. A substantial body of evidence shows that these transitions frequently occur in unplanned and/or crisis circumstances. This evidence relates to people with intellectual disabilities living in the family home (Grey et al., 2020: McCausland et al., 2021; Nankervis et al., 2011; Ryan et al., 2014; Taggart et al., 2012) and in community residential accommodation, with people with intellectual disabilities and behaviours that challenge others at particular risk of community placement breakdown (Bigby et al., 2011; Broadhurst & Mansell, 2007; Philips & Rose, 2010). In the UK, two‐thirds of adults with intellectual disabilities live with their families, typically parents (NICE, 2018b). Older people with intellectual disabilities and behaviours that challenge others are likely to face particular difficulties and stresses. The death of a family member, particularly the main caregiver, can trigger complicated grieving and the need for crisis intervention (Dodd et al., 2005; MacHale & Carey, 2002), in part because parental loss is often accompanied by further losses, including the loss of home (Karavella, 2013). That many older people with intellectual disabilities remain unknown to services is likely to further exacerbate the crisis nature of transition from the family home (NICE, 2018b).

While the above evidence underlines a need for transitions for older people with intellectual disabilities and behaviours that challenge others to be carefully considered and executed, little is known about how to do this. NICE guidance for England relevant to people with intellectual disabilities and behaviours that challenge others [NG 93] advises on the need for transition planning and personalised care, but makes no recommendations around issues relating to older people with intellectual disabilities (NICE, 2018b). Other NICE guidance for the care and support of older people with intellectual disabilities [NG 96] stresses the need for person‐centred planning and review, but does not address behaviours that challenge others (NICE, 2018a). Except for mention of the use of ‘hospital passports’, NICE guidance on transition between care settings [NG 27] does not include recommendations concerning people with intellectual disabilities (either with or without behaviours that challenge others) (NICE, 2015).

To address this evidence‐gap, we reviewed the literature on transition‐related health and social care experiences, service interventions and resources relevant to older people with intellectual disabilities and behaviours that challenge others. In this review, we present the findings according to key themes characterising these experiences, interventions and resources. In so doing, we identify core learning for how the transitions of older people with intellectual disabilities and behaviours that challenge others can be most effectively planned and undertaken to fit with their needs and preferences. Importantly, learning addresses how unplanned and otherwise inappropriate transitions can be avoided. The review was undertaken as part of a wider research project (based in England) evidencing the support for older people with intellectual disabilities and behaviours that challenge others, and their family carers.^1^

## 2 METHODS

Our review was intentionally inclusive and exploratory, designed to capture a broad range of evidence. We therefore undertook a scoping review, enabling the systematic synthesis of evidence from diverse sources according to its nature, features, and findings/outcomes (Peters et al., 2015). As the review constituted the early stage of a broader study, we utilised a rapid review methodology (Tricco et al., 2017). Rapid reviews are a pragmatic and robust approach to evidence generation (Langlois et al., 2019; Pluddemann et al., 2018). In order to reduce the potential for bias, we adhered to what Pluddemann et al. (2018) describe as ‘additional steps’ (p. 202) beyond the minimum required. As such, although our review was, necessarily, expedited, in essential areas (e.g., publication of protocol, search of multiple datasets, and inter‐rater involvement at all stages) it adhered to standard procedures for systematic reviewing. Table 1 outlines our approach.

**TABLE 1:** Outline conduct of our rapid scoping review

Guidance used re: Scoping review	Guidance used re: Rapid review	Specific points of impact re: Rapid approach
Preferred Reporting Items for Systematic reviews and Meta‐Analyses extension for Scoping Reviews (PRISMA‐ScR) Checklist (Tricco et al., 2018).	**S**elec**T**ing **A**pproaches for **R**apid **R**eviews (STARR) (Pandor et al., 2019) decision tool to help make broad decisions concerning the overall review process. The Oxford Centre for Evidence Based Medicine (Pluddemann et al., 2018) and the World Health Organisation (WHO) (Tricco et al., 2018) to help make decisions concerning specific methods/techniques.	Use of limited priority databases. Limited searching for evidence, for example, no citation searching or contact made with experts beyond members of the project team. Limited searching for evidence not immediately available; if not available within a one‐month period, such evidence was recorded as ‘missing’. No critical appraisal undertaken.

Throughout the review we consulted with members from our two expert advisory groups: a professional group, which included health and social care professionals (*n* = 3), policy‐makers and consultants (*n* = 7), commissioners (*n* = 2), service providers (*n* = 3) and a patient and public involvement (PPI) adviser (*n* = 1); and a PPI advisory group, which included one person with intellectual disabilities and her support worker, two parent carers and one sibling carer. Advisors were all invited to contribute to these groups on the basis of their knowledge, expertise and experience in supporting older people with intellectual disabilities and behaviours that challenge others, and/or growing older themselves. While there were more people with intellectual disabilities involved in the two groups, they agreed amongst themselves to ‘play to their strengths’ and focus time and energy on particular strands of project activity. Thus, one person opted to work on the review, while others focused their attention on supporting empirical work packages. Both groups contributed ideas, discussed ongoing findings, and helped to ensure clarity and relevance of analysis. Specifically, we liaised with the groups to finalise our search strategy; to consider the initial full screen returns; and to discuss our preliminary data analysis. The groups therefore contributed to analytic thinking regarding the codes and development of key themes.

### 2.1 Review question and objectives

#### 2.1.1 Research question

What are the health and social care needs, experiences, service interventions and resources of and for older people with intellectual disabilities and behaviours that challenge others as they move to different care contexts^2^ in the UK?

#### 2.1.2 Research objectives

1. Identify relevant UK evidence according to key features such as: nature, focus, content, target population, design, methodology and findings/outcomes;
2. Systematically integrate this evidence for the health and social care needs, experiences, service interventions and resources of and for older people with intellectual disabilities and behaviours that challenge others as they transition to different care contexts;
3. Use the learning delivered by (2), to consider the status of transition‐related care and support for older people with intellectual disabilities and behaviours that challenge others, drawing out implications for how this care and support might be most effectively planned and undertaken to fit with their needs and preferences.

### 2.2 Eligibility criteria

We included published and unpublished (grey) literature, including research articles, reports, and guidance. Within the published research we included primary (using quantitative, qualitative and mixed methods) and secondary (e.g., review) level evidence. To enhance the relevance of our review findings, we included evidence made available after 2001, to coincide with the publication of the Government's *Valuing People* White Paper for England and Wales (Department of Health, 2001). *Valuing People* included an explicit focus on the needs of older people with intellectual disabilities and on the needs of people with behaviours that challenge others. Although health and social care policy and funding is devolved in the UK, there have been calls for greater sharing of knowledge and ‘lessons learned’ across the four nations, given the same constraints that each face with regards to the UK structure of taxes and benefits (Bell, 2010). With this in mind, our review includes relevant material from across the UK, in order to inform our wider research project, which is located in England.

Using the population, concepts and context (PCC) framework (Peters et al., 2020), our inclusion criteria were:

- Published/made available in English after 2001
- Concern older (40+)^3^ adults with intellectual disabilities and behaviours that challenge others resident in the UK
- Concern the health and social care needs, experiences, service interventions and resources of and for these older adults transitioning to different care contexts, for example, from family care to service care; from one context of service care to another (e.g., supported living to residential/nursing home care); and, from one context of family care (e.g., parent‐led) to another (e.g., sibling‐led).

We excluded discussion papers, position papers, expert opinion pieces, editorials and study protocols, as we were interested in the nature of and findings of evidence, which could be used to draw conclusions regarding our phenomena of interest.

### 2.3 Information sources

The development of search strategies and database searches were undertaken with the support of a subject specialist librarian. An initial set of potential databases were reviewed using ‘Healthcare Databases Advanced Search’ (part of NHS Evidence), as a means of determining those (academic and grey literature) most likely to yield relevant evidence. We then identified and searched the following priority electronic databases: Cumulative Index to Nursing and Allied Health Literature (CINAHL) (Ebsco); Healthcare Management Information Consortium (HMIC) (Ovid); NHS Evidence (NICE); Scopus (Elsevier); Turning Evidence Into Practice (TRIP); Web of Science (WoS) (Clarivate); Google (first five pages); and, Google Scholar (first five pages).

We generated search terms (words and phrases, including synonyms and terminology variations), combined using the Boolean operators ‘and/or’ and appropriate truncation and phrase symbols to form initial search strategies, which we piloted against selected key databases. On the basis of the insights gained concerning the sensitivity and specificity of our terms, we confirmed our final search strategies to be used for each database, as well as the Google search strings, limited by file type (PDF) (see Online Appendix 1 for all database search strategies). The reference lists of included evidence identified from the database searches were hand searched. In addition, we used the expertise of the research team and project advisory group to identify other potentially relevant evidence.

### 2.4 Selection of sources of evidence

After de‐duplication by the specialist librarian, electronic search datasets were imported into Excel. Using titles and abstracts (where available), all records were screened by Researcher One, with Researchers Two, Three and Four also independently screening a selection. Any discrepancies were discussed between Researcher One and the other three researchers as appropriate, and in a majority of cases, a final decision reached, in all cases without recourse to a third researcher. In a minority of cases, a decision could not be made until additional information, unavailable in either the title or abstract, was obtained. This process enabled the exclusion of evidence that clearly did not meet the inclusion criteria and identified evidence for full text review.

All full text records were read by Researcher One, with Researchers Two, Three and Four also independently reading a selection. To reduce the potential for bias and promote transparency and consistency in decision‐making, a standardised tool was used (Online Appendix 2). Discrepancies were discussed between Researcher One and the other three researchers as appropriate, and a final decision reached. Evidence excluded on the basis of full text review was recorded, including the reasons for exclusion. In cases where evidence was not immediately available, we attempted to source it using various means (e.g., contacting relevant authors). Given time constraints, if not available within a one‐month period, evidence was recorded as ‘missing’.

### 2.5 Data extraction

A data extraction form was developed, piloted on three sources of evidence selected to ensure variation in focus and content, and a final version (Appendix 3) used to extract data from included evidence. Data extraction was led by Researcher One; all completed forms were shared amongst the research team as a means of checking for gaps and inconsistencies.

### 2.6 Critical appraisal of individual sources of evidence

The conduct of critical (quality) appraisal in scoping and rapid reviews is generally considered optional (Peters et al., 2020; Stevens et al., 2018; Tricco et al., 2017). For scoping reviews, the central issue is inclusion of many types of evidence (Peters et al., 2020), some of which are not amenable to quality appraisal (e.g., user resources). For rapid reviews the central issues are: lack of/limited availability of information on which to base quality assessment decisions (Tricco et al., 2017) and time available to complete the review, including in respect of chasing up missing information (Langlois et al., 2019). Given the variety of included evidence and the project time‐plan, we took a pragmatic decision not to undertake critical appraisal. However, we did consider how included papers framed the concept ‘behaviours that challenge others’ and the extent to which the research took a critical stance towards this concept. In particular, we were alert to papers that presented a medicalised perspective on behaviours that challenge others, and considered how this may have impacted upon the findings presented.

### 2.7 Synthesis of findings

Alongside primary and secondary empirical research findings, evidence included non‐research case studies, and resources providing information and guidance relevant to older (40+) people with intellectual disabilities and behaviours that challenge others. Such diversity necessitated a flexible approach to bringing together the evidence in its entirety. Key characteristics of included evidence were summarised in a table of characteristics (Table 4). Using the evidence included in the table, we identified patterns and trends in the volume, focus and content of included evidence, as a basis of narrative comment in the section 4.

The *findings of* included evidence were integrated using a narrative approach (Popay et al., 2006; Ryan, 2013). An iterative process of reviewing the entirety of the research evidence allowed us to identify patterns in what the evidence was suggesting, however derived and expressed, which we captured in a series of themes and constituent sub‐themes. The process was led by Researchers One and Two, with sustained involvement of Researchers Three, Four, Five and Six from an early stage, and involvement of the advisory groups once an initial thematic draft had been developed (Table 2).

**TABLE 2:** Author/advisory group contribution to development of themes

Stage	Involvement of research team/advisory group	Outcome
1. Data extraction	Data extraction led by Researcher One. Forms distributed to Researchers Two, Three & Four who checked content against evidence in included articles.	Data extraction forms confirmed as accurate record of original data.
2. 1st draft—pulling together of data into preliminary categories (codes) of evidence. Undertaken by Researchers One & Two.	1st draft sent to Researchers Three & Four, who provided feedback on fit with original data e.g. is all of the data captured?; does the coding of the data ‘make sense’ in terms of included content?	Provisional 2nd draft.
3. Provisional 2nd draft comprehensively reviewed, based on revisiting of original data, suggestions made by Researchers Three & Four and beginnings of more conceptual thinking. This process resulted in a pulling together of the codes and their content to produce descriptive themes and sub‐themes. Undertaken by Researchers One & Two.	2nd draft sent to Researchers Three, Four, Five & Six, who provided feedback on content, particularly concerning the relevance/appropriateness of the descriptive themes, for example, do they capture the essence of their content?; For example are the descriptive themes appropriate in terms of the focus of the review?	Provisional 3rd draft.
4. Provisional 3rd draft reviewed, based on suggestions made by Researchers Three, Four, Five & Six, and continuing conceptual thinking. This process resulted in further development of themes/sub‐themes (e.g. re‐framing of existing themes, and attendant movement of content between themes) to produce analytical themes that were directly relevant to the focus of the review (i.e., transitions). Undertaken by Researchers One & Two.	3rd draft sent to Researchers Three, Four, Five & Six and project advisory team, who provided feedback on content and its overall sense and insight provided. Feedback included that addressing: conceptual issues, for example, ensuring that the themes/sub‐themes ‘made sense’/were meaningful in terms of the focus of the review how content needed to be amended/augmented/re‐presented to improve clarity how content could be reduced to fit with word count of journal intended for submission implications of findings.	Provisional 4th draft.
5. Provisional 4th draft reviewed, based on suggestions made by Researchers Three, Four, Five & Six and project advisory group, and continuing conceptual thinking. This process resulted in re‐organisation/re‐framing of existing themes and their content to better reflect meaning and relevant to the focus of the review. Undertaken by Researchers One & Two.	4th draft sent to Researchers Three, Four, Five & Six who provided feedback. At this stage, suggestions from team focused on ensuring that: the draft adequately reflected feedback on 3rd draft the developed themes were conceptually adequate in terms of capturing core meaning of their content and relevant to the focus of the review the overall ‘narrative’ fitted together as a coherent whole.	Provisional 5th draft.
6. Provisional 5th draft reviewed based on feedback. Undertaken by Researchers One & Two.	5th draft sent to Researchers Three, Four, Five & Six and project advisory group for final review. Feedback invited as per Stage 4.	Provisional 6th draft.
7. Provisional 6th draft reviewed based on feedback. Undertaken by Researchers One & Two.	Final review also included focus on grammar, style, and overall presentation.	7th and final draft—sent to all.

Our aim in the analysis was to interpret, rather than merely describe, the original (author‐generated) findings. Thus, we sought to generate new conceptual understanding, set out as analytical themes (as distinct, e.g., from merely collating the evidence into descriptive categories). To do so, data were analysed deductively and inductively. Deductively, we took the focus of the review—transitions—as our point of entry into the data. Practically, this meant that the question ‘What does this evidence mean for transition for older people with intellectual disabilities and behaviours that challenge others?’ underpinned all interpretation. Inductively, we followed a three‐stage process (Table 3), meaning that the development of our final themes was based primarily on the peer reviewed articles from our included evidence.

**TABLE 3:** Analytical process of theme development

Stage	Analysis	Outcome
Stage 1: Development of coding framework	Article by article development of codes, which reflected directly the meaning and content of the author generated findings.	Equivalence of coding/categorisation of data across the collective body of findings.
Stage 2: Development of descriptive themes/sub‐themes	Iterative review of all codes to identify those that clustered together in terms of their meaning to produce ‘descriptive themes’. Each theme given a name that descriptively summarised its content/focus.	Equivalence of ‘descriptive themes’ across the collective body of findings.
Stage 3: Development of analytical themes/sub‐themes	Iterative review of descriptive themes, including their individual codes and associated segments of data, in terms of their meaning/relevance to the focus of the review (i.e., transitions) to develop analytical themes/sub‐themes. Extended inductive process of interpretation, involving movement between the descriptive themes, their constituent codes and associated bodies of data, and developing analytical themes. Shared research team/project advisory input enhanced the reliability of the final analytical framework, as the full possibilities for analytical insight were levered.	Set of conceptually relevant analytical themes/sub‐themes.

## 3 FINDINGS

### 3.1 Search results

**FIGURE 1:**
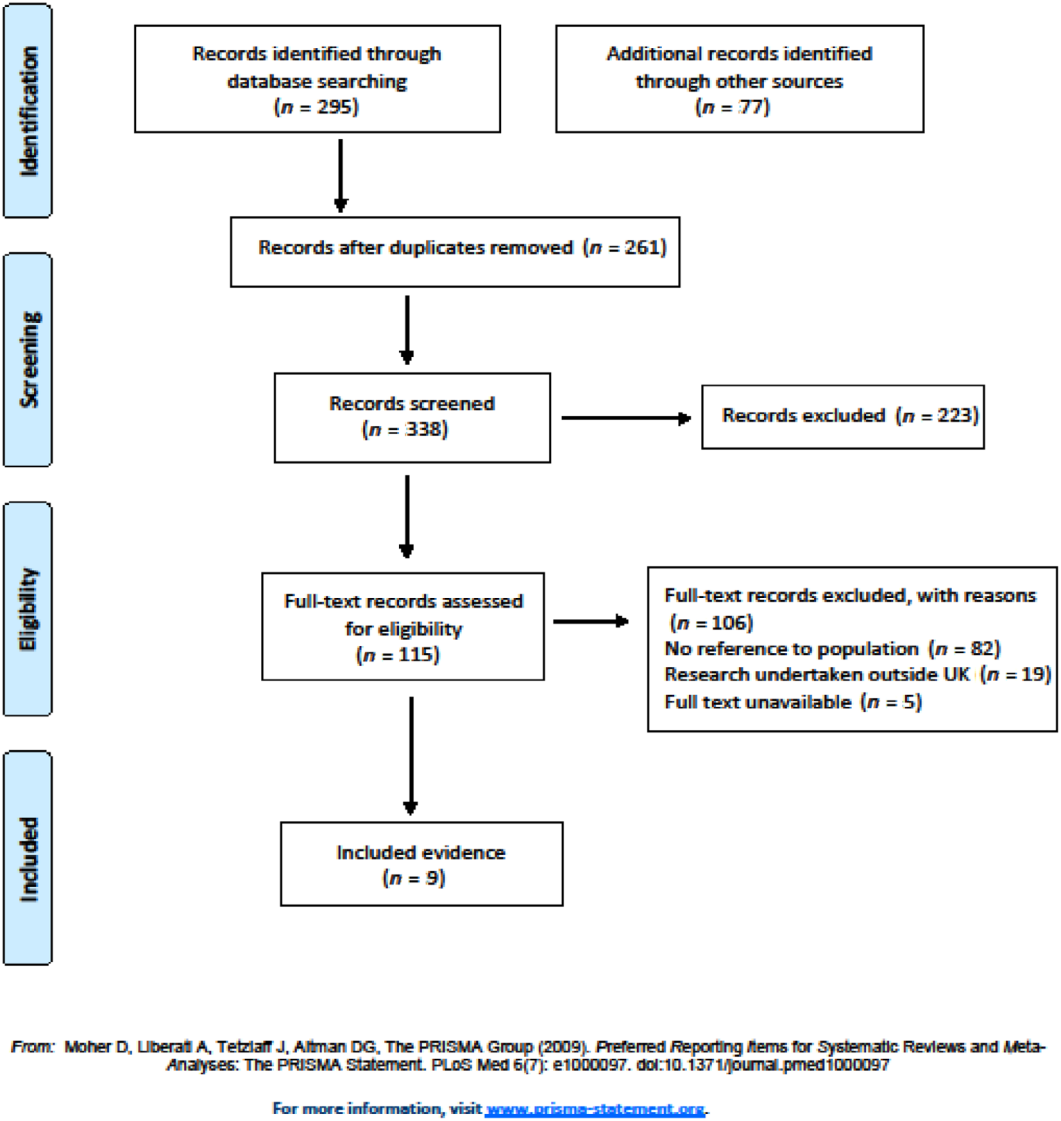
Search flow chart

Database searches yielded 261 returns, of which 223 were excluded on the basis of initial screening (using titles/abstracts). Of the remaining 38 read in full, 32 were excluded and six were identified for inclusion. A total of 37 returns were identified from the reference lists of database included articles, all of which were read in full. Of these, one article was included. A total of 40 items of evidence were identified by the research team and/or our advisory group, all of which were read in full. Of these, two were included. Therefore, a total of nine items of evidence were included in our review (Figure 1).

### 3.2 Study characteristics

Our evidence included five peer‐reviewed journal articles, three reports and one resource. Two of the journal articles reported single client case studies and three reported empirical research. One report was based on empirical research, one on a rapid review of evidence and no information on the source of included evidence was provided for the third. No information was provided on the source of included evidence for the resource. Five sources of evidence referred to England, two referred to Wales, one referred to evidence published in English and no relevant information was provided for the remaining source of evidence. Table 4 summarises the characteristics of included evidence.

**TABLE 4:** Characteristics of included evidence

Authors/date of publication	Title	Type of evidence	Stated aim/purpose	How evidence assembled	Population	Definition or examples of ‘behaviour that challenges others’	Country	Main findings
Bissell et al. (2005)	The experience of a man with severe challenging behaviour following resettlement from hospital: a single case design.	Peer‐reviewed journal article	To investigate the effectiveness of a behavioural intervention in the management of problem behaviours.	Single client case study, based on clinical psychology caseload notes.	Single client (55 year old male) with severe intellectual disabilities. Other conditions: epilepsy.	Examples of behaviour provided: shouting, screaming, destruction of environment, smearing faeces, physical aggression to residents and staff.	England	Significant decrease in challenging behaviour after implementation of guidelines. Effective dental treatment produced further reduction in challenging behaviour.
Forrester‐Jones (2019)	Confronting a looming crisis. People with learning disabilities or autism and their carers getting older.	Report	To explore the experiences of older family carers as they continue to care for their older adult relatives with intellectual disabilities.	Research—qualitative; data collection = interviews with older family carers; data analysis = Interpretative Phenomenological analysis (IPA).	Carers (50+) of adults (*n* = 16; average age = 45; 50% over age of 50; gender not stated, but mixed gender) with mild to severe intellectual disabilities. Other conditions: Smith‐Magenis Syndrome; Down Syndrome (*n* = 5); cerebral palsy (*n* = 2); autism (n = autism). Two participants described as displaying behaviours that challenge others.	None provided.	England	Family carers' avoidance of future planning unintentionally thwarts opportunities for older people with intellectual disabilities to learn about options for transition. Issue of choice by individuals not straightforward in all situations, with possibility of conflict with family carers. Reduced effectiveness of social work support due to frequent staff turnover. Early and proactive professional involvement in supporting a ‘whole family’ approach to transition planning is required. Lack of/delays to needs assessment of people with intellectual disabilities, and lack of functional assessments or positive behaviour support plans. People's transition ‘stifled’ because of carers' aversion to sending adult family member to accommodation they consider unsuitable.
Hubert and Hollins (2010)	A study of post‐institutionalised men with severe intellectual Disabilities and challenging behaviour.	Peer‐reviewed journal article	To examine the after‐effects of moving from institutional care to small group homes amongst adults with challenging behaviours.	Research—ethnography undertaken longitudinally (6 years).	Men (*n* = 20; age range 29–46) resident in institutional hospital, with profound to severe intellectual disabilities. Other conditions: majority with autistic spectrum disorder and ‘concomitant’ (p. 190) mental health problems.	Examples of behaviour provided: physical aggression, self‐injury, taking off clothes, ripping up clothes, spreading urine and faeces, eating unsuitable objects.	Not stated	Participants' lives improved materially after taking up residence in new homes, but they continued to experience social exclusion and denial of individual identity and autonomy. Few fundamental changes in professional and social attitudes towards them.
Leaning and Adderley (2015)	From long‐stay hospitals to community care: reconstructing the narratives of people with learning disabilities.	Peer‐reviewed journal article	To describe the journey taken by a man from institutional care to community living.	Single client case study, based on clinical psychology caseload notes.	Single client (62 year old male) with severe and profound intellectual disabilities. Other conditions: autistic spectrum disorder.	Examples of behaviour provided: aggression, self‐injury, biting, hitting, shouting.	England	Over extended period of time, the man was resettled in community. Involved: clinical psychologist's intense support and advocacy; wider psychology team support, including development and implementation of PBS plan; training for home care staff team and development of transition and subsequent care plan; and, involvement of the man's family and the man himself, with appropriate support. Process complicated by need to adhere/pass numerous legal requirements.
Perry et al. (2011)	Resettlement Outcomes for people with severe challenging behaviour moving from institutional to community living.	Peer‐reviewed journal article	To evaluate the quality of life consequences of resettlement from an intellectual disability hospital to new purpose‐built accommodation.	Research—undertaken longitudinally (12–18 months); data collected on quality of care and lifestyle indictors.	Adults (*n* = 18; 13 male/6 female; age range 36–67 years; mean 47 years) with intellectual disabilities, ranging from lower to upper scores on the Adaptive Behaviour Scale (ABS, Nihira et al., 1993). Other conditions: epilepsy (*n* = 4); autism (*n* = 3); mental ill‐health (*n* = 7).	Participants classified using the Aberrant Behaviour Checklist (Aman & Singh, 1986): irritability, lethargy, stereotypy, hyperactivity, inappropriate speech.	Wales	Quality of care and quality of life outcomes were generally equivalent or superior to previous hospital levels. Improvement over time was demonstrated in respect of greater family contact and reduction in staff‐reported challenging behaviour.
Perry et al. (2013)	Adults with intellectual disabilities and challenging behaviour: the costs and outcomes of in‐ and out‐of‐area placements.	Peer‐reviewed journal article	To compare the costs and outcomes of in‐ and out‐of‐area placements for people with intellectual disabilities and challenging behaviour.	Costs, quality of care and quality of life outcomes.	Adults (*n* = 76; 48 male/28 femal; mean age of men = 46/mean age of women = 35) with intellectual disabilities, ranging from lower to upper scores on the Adaptive Behaviour Scale (ABS, Nihira et al., 1993). Other conditions: mental illness (28.9% in‐area and 15.8% out‐of‐area); autistic spectrum disorder (47.4% in‐area and 44.7% out‐of‐area).	None provided.	Wales	There was a mixed pattern of quality of care and quality of outcome advantages between the two types of setting. In‐area placements had a greater number of advantages than out‐of‐area placements. Out‐of‐area placements had lower total costs, accommodation costs and daytime activity costs.
Sense (2018)	Decisions to make, steps to take. A guide to planning long‐term care and support for disabled adults and their families. A Sense Toolkit.	Resource—sets out information, guidance, and tools	To provide information for people with intellectual disabilities and their families to start making plans for the future.	Non‐research; No information provided.	People with intellectual disabilities; family carers of people with intellectual disabilities.	None provided.	Not stated: Sense is a UK based organisation	Comprehensive, easy‐to‐read guide, setting out main options available, legal rights possessed, and key decisions that need to be mad regarding making plans for the future care for people with intellectual disabilities.
Slevin et al. (2011)	A rapid review of the literature relating to support for people with intellectual disabilities and their family carers when the person has behaviours that challenge and/or mental health problems; or they are advancing in age.	Report	1. What services and support do people with intellectual disabilities who display behaviours that challenge and their caregivers require to meet their needs? 2. What services and support do older people with intellectual disabilities and their caregivers require to meet their needs?	Rapid review, using a framework adapted from the NHS Centre for Reviews and Dissemination (CRD, 2009) and the Rapid Review Methodology (NHS Wales 2006).	People with intellectual disabilities who display behaviours that challenge; Carers of people with intellectual disabilities who display behaviours that challenge. Older people with intellectual disabilities; Carers of older people with intellectual disabilities. Other conditions: no information provided but behaviours that challenge others is ‘inclusive of mental health problems’ (p. 9).	Working definition of behaviours that challenge others provided (p. 9): ‘…severely challenging behaviour refers to culturally abnormal behaviour(s) of such an intensity, frequency or duration that the physical safety of the person or others is likely to be placed in serious jeopardy, or behaviour that is likely to seriously limit use of, or result in the person being denied access to ordinary community facilities’ (Emerson (1995, p. 44)'.	Only studies published in English included	People whose behaviour challenges Support should be based on the use of resources to maintain the person in their own home, if this is their wish. Identified interventions and services (e.g., PBS, use of community specialist teams, short breaks, teaching and supporting caregivers) were found to be successful in doing so. Appropriate day opportunities also essential, but have not been adequately researched, as is family support to allow people with intellectual disabilities and behaviours that challenge and their family to lead fulfilling lives. Medication highly used, but behavioural management should be pursued as appropriate. Interventions most likely to be effective when delivered via a family support and education approach, in partnership with formal carers. Active support appears a promising approach. Specialist community teams are a highly effective service. Specialist assessment and treatment units can provide a useful service, but admission should be for a short period, with aim of return to the community. To this end, a model that provides combined specialist support services is recommended. Evidence suggests limited use of full range of mental health services, suggesting deficits in terms of their accessibility and value for people with intellectual disabilities and behaviours that challenge. Older people with intellectual disability Most older people with intellectual disabilities and behaviours that challenge wish to continue to live in their family home, and ageing family carers want to continue caring. However, lack of future planning persists. Ageing people with intellectual disability may face same range of health‐related issues as others earlier in their lives. In addition, there are higher rates of some conditions (e.g., dementia). Medications that can help are seldom offered. Appropriate health screening is needed, but scarce evidence that this happens, or that detected health problems are properly investigated and treated. Positive mental health is promoted by such health‐improving behaviours, continuing to remain active, and having a meaningful and valued life. Relevant training is required for frontline staff to develop skills to provide appropriate care, both in supported living arrangements or in partnership with family carers. Evidence suggests that facilities geared towards the needs of older people with intellectual disability are at best scarce and at worst non‐existent. Nursing or residential placement should not be the ‘go‐to’ option; a range of intermediate care facilities should be used as appropriate to promote opportunity to return to their home. It should not be assumed that because a person is a particular age that they may not be able to return to their normal home or residence.
The Housing and Support Partnership, 2011	Planning and Commissioning housing for people with learning disabilities. a toolkit for local authorities.	Report	Resource (‘toolkit’)	Non‐research; No information provided.	Local authorities responsible for the planning/commission‐ing of housing for people with intellectual disabilities.	None provided.	All references made in respect of England, and all examples drawn from England	Toolkit—to assist local authorities/their partners to plan for the housing requirements of local people with intellectual disability. Set out in two parts. Planning – the steps likely to be required to plan for a wider choice of housing options. Delivery – the steps and actions likely to be required to commission a choice of housing options. Each section contains a checklist series of questions and suggestions for possible approaches to make progress. Toolkit designed so that each section can be used independently or sequentially.

Our analysis generated four main themes and eight sub‐themes. The first main theme ‘Transition over the long‐term: laying the necessary foundations’ addresses factors that work, long‐term, for and against successful transition, captured in three sub‐themes: ‘Opening up choice: support to enable meaningful expression of needs and preferences’; ‘Promoting independence, social skills and preparedness’; and ‘Making choice a reality: delivering appropriate accommodation’. The second main theme ‘Avoiding the need for unwanted / inappropriate transition’ deals with factors that can both help maintain residence and prevent crisis moves, captured in three sub‐themes: ‘Optimising health and social care’; ‘The importance of staff training’; and ‘Specialist community teams for people with behaviours that challenge others’. The third main theme ‘At the point of transition: making it work’ deals with the factors that promote the potential for successful transition, captured in two sub‐themes: ‘Specialist support in making transition happen’; and ‘Front‐line staff skills and attitudes’. The final main theme ‘An absence of targeted resources’ deals with the limited information and guidance that is currently available to aid adults with intellectual disabilities and behaviours that challenge others to navigate the complex world of transition (Table 5).

**TABLE 5:** Main themes and sub‐themes by supporting evidence

Main theme	Sub‐theme	Supporting evidence
Transition over the long‐term: laying the necessary foundations	Opening up choice: support to enable meaningful expression of needs and preferences	Slevin et al. (2011) and Forrester‐Jones (2019)
	Promoting independence, social skills and ‘preparedness’	Slevin et al. (2011)
	Making choice a reality: delivering appropriate accommodation	Slevin et al. (2011), Forrester‐Jones (2019), Housing and Support Partnership (2011) and Perry et al. (2013)
Avoiding the need for unwanted/inappropriate transition	Optimising health and social care	Slevin et al. (2011) and Forrester‐Jones (2019)
	The importance of staff training	Slevin et al. (2011) and Perry et al. (2011)
	Specialist community teams for people with behaviours that challenge others	Slevin et al. (2011)
At the point of transition: making it work	Specialist support in making transition happen	Leaning and Adderley (2015)
	Front‐line staff skills and attitudes	Hubert and Hollins (2010) and Bissell et al. (2005)
An absence of targeted resources		Housing and Support Partnership (2011) and Sense, 2018

### 3.3 Transition over the long‐term: Laying the necessary foundations

We consider our inclusion of long‐term factors that work for and against successful transition as valid in that, irrespective of their proximity to the point at which transition occurs, the evidence demonstrates their potential to have a tangible impact on its effective discharge.

#### 3.3.1 Opening up choice: Support to enable meaningful expression of needs and preferences

Transition planning involves proactive planning for circumstances in which people with intellectual disabilities are no longer able to remain in their primary place of residence—typically, the family home (Slevin et al., 2011). Collectively, evidence showed limited planning by ageing family carers and adults with intellectual disabilities. Slevin et al. (2011) cite the findings of several studies where a majority or sizeable minority of participants had not made future plans. The same pattern of limited future planning is reported by Forrester‐Jones (2019), who proposes that avoidance by family carers can thwart opportunities for adults with intellectual disabilities to learn about available options for transition. Highlighting a lack of consistent social work support, Forrester‐Jones (2019) suggests this contributes to deficits in future planning as it prevents the development of informed (on the part of social work staff) and trusting (on the part of families) relationships.

To open up choice for the people with intellectual disabilities, professional involvement in supporting a ‘whole family’ approach to transition planning is advocated by Forrester‐Jones (2019) and Slevin et al. (2011), with the latter stressing the need for planning to start early, before problems associated with ageing in both people with intellectual disabilities and family carers become manifest. They also recommend regular re‐visiting of plans, so that changes in circumstances and needs can be considered and plans amended accordingly. Slevin et al. (2011) stress that concerted efforts be made to enable people with intellectual disabilities to communicate their needs and preferences in line with a person‐centred approach. This includes taking the time to facilitate meaningful expression, using different modes of communication. In this context, Forrester‐Jones (2019) found little evidence of innovative practice. Accordingly, despite what the author considers ‘huge advances in this area’ (p. 60) including the development of alternative and augmentative communications such as Talking Mats, all of the needs assessments described in her study used traditional paper‐based processes.

#### 3.3.2 Promoting independence, social skills and ‘preparedness’

Given that older people with intellectual disabilities who live at home may have limited experience of living more independently, Slevin et al. (2011) identify a need to prepare them for a future life in which the immediacy or intensity of a home‐based support network is no longer available. In this context, the authors identify a role for a range of community services, detailing how they can contribute to such preparatory work (Table 6).

**TABLE 6:** Service support to promote successful transition to independent living

Service	Support
Day centres	Could play a greater part in preparing people with intellectual disabilities, particularly with relevant skill development; advice and support could also be given to family carers concerning how they might enable their family member to practice and develop these skills.
Community support services	Remit could be extended to include a formal role in assisting with planning and preparing for the future.
Short/respite breaks	Could be promoted as having a developmental component, to promote the acceptability of separation and practically move the person towards independence as part of a broader life planning exercise. For this to work, short break services would need to include an explicit skills development component, linked with the same provided to family carers to enable continuity of skill development in the family home.

Slevin et al. (2011) make an important broader point; resource constraints mean that social work services tend to concentrate on priority need, so that people with intellectual disabilities who live at home are less likely to meet the threshold for active social work involvement. Commissioners need to be aware of the longer‐term detrimental impact of this approach, including future financial outlay. Although Slevin et al. (2011) do not explicitly address these issues in respect of people with intellectual disabilities and behaviours that challenge others, the points they make are equally, even especially, pertinent to this population and their family carers.

#### 3.3.3 Making choice a reality: Delivering appropriate accommodation

If people with intellectual disabilities and behaviours that challenge others are to transition successfully, then appropriate accommodation needs to be available. Availability of such accommodation was shown to have a direct impact on carers' willingness to consider the possibilities for, and be content with, transition taking place (Forrester‐Jones, 2019; Slevin et al., 2011), with the former describing transition as being stifled because of carers' aversion to sending their family member somewhere they considered unsuitable. To promote accessibility to the full range of *appropriate* accommodation, the evidence prioritises the responsibility of service commissioners to proactively plan for its delivery. Such planning requires detailed knowledge of the needs of the local population, and how these are likely to change over time, premised on robust databases of ageing family carers and older people with intellectual disabilities (Slevin et al., 2011). This includes specific information relating to ageing, (risk of) dementia and behaviours that challenge others. Slevin et al. (2011) offer guidance on how databases can be compiled to ensure the inclusion of older people with intellectual disabilities and their family carers otherwise unknown to services. Although not exhaustive, the guidance demonstrates the dividends to be paid by creative thinking, capitalising on local‐level opportunities.

Our review found a number of resources designed for use by commissioners in the planning and development of housing for people with intellectual disabilities. Although not specifically targeted at older people with intellectual disabilities and behaviours that challenge others, some of the resources are relevant to that population. A ‘toolkit’ published by The Housing and Support Partnership (2011), provides guidance on how to deliver (in terms of both the planning and delivery) a full range of accommodation, with the specific aim of enabling more people with moderate to severe intellectual disabilities to live in their own homes, in their local community. It is beyond the scope of this review to present detailed content, but one example of ‘good practice’ is worth describing for the lessons regarding the possibilities for, and requirements associated with, successful transitioning. This concerns a London housing project, which involved joint working across eight local authorities and enabled people (including older people with intellectual disabilities and behaviours that challenge others) to move out of long stay assessment and treatment units and other institutional settings. Extensive multi‐disciplinary and multi‐agency collaboration underpinned the development of person‐centred plans and the subsequent work to translate them into individual service designs. In terms of day‐to‐day living, people were provided with a range of assistive technology to support independence. The guidance recommends this project as demonstrating meaningful, multi‐agency partnership to arrive at improved outcomes for people with complex needs and/or behaviours that challenge others.

Other evidence included in our review provides useful insights into the nature of appropriate accommodation to support successful transitioning for older people with intellectual disabilities. In terms of type of suitability, Slevin et al. (2011) argue against moving older people with intellectual disabilities into mainstream residential/nursing facilities (which happens, typically, because of a shortage of specialised accommodation and support) on the grounds that staff working there are unlikely to have relevant support skills. In relation to quality of life, the issue of maintaining older people's links with their family and wider community is critical. This is especially the case regarding the use of ‘out‐of‐area ‘placements, which create a range of difficulties for people with intellectual disabilities (e.g., no longer able to maintain familial and wider social connections). In terms of the implications of this evidence for effective transition, a model of service provision that prioritises ‘ageing in place’ is suggested, encompassing both proactive and reactive strategies (e.g., emergency plans) (Slevin et al., 2011).

Based on the evidence included in their review, Slevin et al. (2011) recommend three main models of provision (Table 7).

**TABLE 7:** Models of service provision

Model of provision	Nature of model/approach
Ageing in place	Older people with intellectual disabilities with dementia remain in the family home with appropriate support and adaptations as necessary. These include: individual early screening, clear diagnostic pathways, improvements to the environment, outreach services, palliative care services, speech and language therapy, respite care, carer education and training.
In place progression	The environment is developed to become increasingly specialised to ensure appropriate care within an intellectual disability setting. This requires staff education and training, waking night staff, environmental adaptations, outreach services, palliative care services, and speech and language therapy.
Referral out	People with intellectual disabilities and dementia move to long‐term (usually nursing) mainstream facilities, with staff supported (e.g., training) to provide appropriate care.

Some of the benefits of remaining in one's local area are demonstrated by the findings of Perry et al. (2013), who compared the costs and outcomes of in‐ and out‐of‐area placements for people with intellectual disabilities and behaviours that challenge others. Although both types of placement had advantages and disadvantages in terms of the outcomes measured, in‐area placements were found to have smaller settings; higher staffing levels; more consistent staff training and support; greater contact with specialist providers; more common procedures for targeted assessment and response to behaviours that challenge others; less frequent use of physical restraint; more frequent reviews of medication for mental health problems or behaviours that challenge others; lower turnover of living companions; more common attendance at daytime social clubs; greater frequency of community activities; more frequent visits from friends; and, lower costs of maintaining contact for families and friends.

### 3.4 Avoiding the need for unwanted/inappropriate transition

If people with intellectual disabilities and behaviours that challenge others are to avoid being moved to unfamiliar and potentially unsuitable settings as they age, there is a need to attend to multiple issues impacting on them, their front‐line professional support workers and their family carers (Slevin et al., 2011). The following sections deal with these prerequisites, as supported by the evidence included in our review. Failure in respect of any leaves people with intellectual disabilities vulnerable to unplanned and possibly inappropriate transition.

#### 3.4.1 Optimising health and social care

Despite policy requirements that needs assessments be undertaken as a means of identifying the care and support needs of people with intellectual disabilities (Department of Health and Social Care, 2014; NICE, 2018a), there is clear evidence of major deficits in the assessment and/or implementation phases. Forrester‐Jones (2019) describes considerable frustration on the part of participants regarding either a lack of, or delays to, needs assessment of their family member. For some, the time lag between the request for a needs assessment and its completion went far beyond the four to 6 weeks specified in The Care Act 2014 (England). In addition, participants reported a failure to adequately implement a needs assessment when undertaken. Forrester‐Jones (2019) also identifies failures in the operationalization of the Integrated Personal Commissioning (IPC) programme, introduced by NHS England in 2014 across nine pilot sites to support people with complex needs to reside at home, avoiding the need for potentially long‐term moves into hospitals/assessment and treatment centres. Although the vast majority of Forrester‐Jones' (2019) participants were caring for a person with intellectual disabilities with additional complex needs, only one was aware of the IPC programme.

Given the known relationship between mental and physical health issues and behaviours that challenge others, the need for a proactive approach to effective healthcare of older people with intellectual disabilities and behaviours that challenge others is particularly acute. Slevin et al. (2011) prioritise annual general practice (GP) health checks as a useful preventative strategy, as they can reveal ongoing and/or changing health needs. That older people with intellectual disabilities have been shown to under‐use health services further underscores the need for proactive engagement (Slevin et al., 2011). Of particular concern is dementia. Slevin et al. (2011) discuss the complex interplay between the degree of intellectual disability and social, environmental and health factors in how dementia presents and is understood by professional and family carers. Although those with an established relationship with people with intellectual disabilities are well placed to recognise even small changes in behaviour, difficulties in diagnosis include the potential for symptoms of dementia to be confused with ‘normal’ ageing, other co‐morbidities, and/or as a manifestation of behaviours that challenge others. For these reasons, Slevin et al. (2011) highlight the need for education and training to be provided on both the recognition of the early symptoms of dementia, as well as the care of people with dementia. In combination with the regular assessment already described, this training is recommended as enhancing the opportunities for early diagnosis, timely therapeutic support and improved prognosis (Slevin et al., 2011). Clearly, all such care and support is central to helping people with intellectual disabilities to remain in their home of choice.

In terms of behaviours that challenge others, based on their included evidence, Slevin et al. (2011) recommend positive behavioural support (PBS) as a first‐line intervention. PBS recognises behaviours that challenge others as a potential response to triggers such as physical or mental health problems, environmental factors, the result of learned behaviour, and/or an inability to communicate effectively other than engaging in such behaviour. Accordingly, behaviour support plans based on this approach should be person‐centred, supportive, practical and sustainable, and relevant to the ‘real world’ of the person with intellectual disabilities and behaviours that challenge others. In the PBS approach, a crucial first step is to undertake a ‘functional analysis’ assessment of behaviours as a way of understanding their causes and how they function for the person involved. In the Forrester‐Jones (2019) study, two participants identified their family member as displaying behaviours that challenges others, and others described behaviours that could be regarded as challenging. However, none recalled having been offered functional assessments or positive behaviour support plans.

#### 3.4.2 The importance of staff training

Given that PBS includes the role of the home environment (both physical infrastructure and people), Slevin et al. (2011) conclude that making relevant changes can be a powerful strategy in reducing behaviours that challenge others. As the authors note, carers (whether professional or family) need appropriate skills and methods of working, premised on relevant education and training, as well as other support, such as professional supervision, team building and stress management. Support for the claim that staff training can play a crucial role in effectively responding to behaviours that challenge others comes from Perry et al. (2011). Their study focussed on the staggered move of 18 older adults with intellectual disabilities and behaviours that challenge others from long‐stay hospital to supported community living. Staff training in PBS was provided, alongside the development of person‐centred plans for all residents. The fact that a reduction in behaviours that challenge others was observed from the outset of the resettlement process, when a majority of participants remained in hospital, leads the authors to prioritise staff training as the operative variable rather than resettlement per se. By the end of the intervention, a significant overall improvement in behaviours that challenge others was observed. That significant increases in family and other community activity also occurred for participants, beginning whilst still in hospital, once again suggests the importance of the training provided to staff in preparation for the move.

#### 3.4.3 Specialist community teams for people with behaviours that challenge others

This section focuses on the service infrastructure within which the interventions outlined above, as well as other relevant care and support, are delivered. Slevin et al. (2011) state there is ‘strong empirical evidence’ (p. 59) of the benefits of community specialist teams in meeting the complex needs of people with intellectual disabilities and behaviours that challenge others and/or mental health problems, drawing particular attention to their contribution to reducing the need for hospital or specialist unit admissions. Generic advantages of specialist teams include their location in the community, accessibility and multi‐professional/multi‐agency approach. Specific advantages for people with intellectual disabilities and behaviours that challenge others is their provision of PBS, reducing admission to hospital or out‐of‐home placements, and delivering improvements to quality of life. Given these advantages, Slevin et al. (2011) recommend that people with intellectual disabilities and behaviours that challenge others and their caregivers (family and professional) should have access to a specialist behavioural team to provide support as required. However, the authors note the limited availability of specialist teams, meaning that ‘few’ (p. 58) are likely to gain access.

### 3.5 At the point of transition: Making it work

This section continues consideration of the collective evidence about factors that are important in promoting or impeding successful transition for older people with intellectual disabilities and behaviours that challenge others. The focus is on factors relating to the immediate run‐up to, point of and period following transition.

#### 3.5.1 Specialist support in making transition happen

In line with the evidence regarding the value of specialist community teams (3.4.3), included studies confirm the benefits of specialist involvement at the point of transition. Leaning and Adderley (2015), for example, report a case study of the move from institutionalised care to community life by a 62 year old man with profound intellectual disabilities and behaviours that challenge others. The case study demonstrates the extended planning and intense support required to effect the move, involving the efforts of a multidisciplinary team, assembled and led by a committed clinical psychologist. Key elements of the transition process included the development of a personalised support plan, training of staff carers in PBS and communication techniques, preparatory exercises for the man, including familiarisation with staff carers, and family involvement in decision‐making throughout. Based on this intensive planning and preparation, the man successfully transitioned to his own flat, accompanied by personalised care that supported his needs. Leaning and Adderley (2015) conclude that transition from long‐stay hospital to community‐based living was achieved because of the development of a different ‘view’ of the man, one which eschewed a strictly behavioural or psychiatric perspective.

#### 3.5.2 Front‐line staff skills and attitudes

In this section we concentrate on the importance of staff skills and attitudes in supporting a successful transition. Hubert and Hollins (2010) examined the impact on the quality of life of adult men with intellectual disabilities, and labelled by services as having long‐term behaviours that challenge others, of transitioning out of an institutional hospital after its closure. Although the lives of all men were found to have improved in material ways after their move, the authors stress that these material changes were only part of a complex set of relevant factors. In terms of staff attitudes, based on their observations over several years, Hubert and Hollins (2010) claim that the most sustained improvement in quality of life was achieved by the men who moved into a campus‐based home, which they link to the approach and commitment of the manager of the home. For the men who moved into two community‐based homes, Hubert and Hollins (2010) suggest a more inconsistent improvement in their quality of life, again linked to the attitude and style of individual managers, of whom there was a frequent turnover, and exacerbated (in House B) by the frequent use of agency staff. Their extended observations of all three homes over many years lead Hubert and Hollins (2010) to conclude that, irrespective of the location of the accommodation, the quality of the men's lives was primarily dependent on the attitude and level of dedication of those in direct charge of the homes and, as a result, on the quality and training of care staff.

These findings are supported by Bissell et al.'s (2005) longitudinal study of the transition of a 55 year old man who, after 44 years in institutional care, was moved to a small group home for adults with intellectual disabilities. Based on their observations conducted over several years, Bissell et al. (2005) conclude that care staff have a critical role to play in observing and identifying the potential causes of behaviours that may be challenging to others (for example, in this particular case, dental pain), and responding accordingly. The authors also identify a role for specialists other than clinical psychologists and behavioural therapists ‐ particularly that of speech and language therapists ‐ in the assessment and response to behaviour that challenges others.

### 3.6 An absence of targeted resources

Our review found no targeted resources to guide older people with intellectual disabilities and behaviours that challenge others, their families, or professionals (both front‐line and planners/commissioners), in planning and decision‐making. We did find some generic resources, some of which included limited relevant content. Most likely as a result of their lack of contemporaneity, we were unable to access the majority of those identified. Table 8 summarises our findings. Identified resources should not be considered exhaustive; as a rapid review, some may have been missed. Where the identified generic resources included content specifically relevant to older people with intellectual disabilities and behaviours that challenge others, we provide some detail.

**TABLE 8:** Identified resources

Resources for professionals responsible for care planning and provision	Access
Sense (2018)—a UK national disability charity that supports people with complex communication needs—published a ‘toolkit’, intended for use by people with intellectual disabilities and their families when making plans for the future. It outlines key decisions that need to be made, sets out the steps to take in making these decisions and includes detailed information on the main options available, resources to support and legal rights possessed in respect of both processes. Notwithstanding its generic ‘pitch’, some of the content is extremely useful for people with intellectual disabilities and behaviours that challenge. This content includes details on NHS Continuing Healthcare Funding, Personal Health Budgets, statutory advocacy provision, and practical resources (templates) to be used to aid planning and decision‐making. www.sense.org.uk	Accessed 30th May 2021
The Housing and Support Partnership (2011) lists: ARC: ARC is a membership organisation, which supports providers of services to people with intellectual disabilities. http://www.arcuk.org.uk	Accessed 30th May 2021
Resources for older PWLD + BTC	
The Housing and Support Partnership (2011) lists: Bath and North East Somerset: ‘My Own Home; guide to housing for people with learning difficulties’—the guide provides advice regarding different housing and support options available, including information about funding for supported living. It is available on tape and CD. http://www.bathnes.gov.uk/BathNES/healthandsocial/helpforadults/adultslearning/supportedliving	Tried and failed to access this resource 30th May 2021
The Housing and Support Partnership (2011) lists: Mencap: factsheet providing information on consent, decision‐making and financial matters. www.mencap.org.uk/displaypagedoc.asp?id=12744	Accessed 30th May 2021
The Housing and Support Partnership (2011) lists: Making Money Easier: a set of guides designed to help people with intellectual disabilities to think about money, banking and planning their lives. http://www.making‐money‐easier.info	Tried and failed to access this resource 30th May 2021
The Housing and Support Partnership (2011) lists: Housing Options: ‘Your Place to Live’—a guide to housing options of people with intellectual disabilities available to download free. www.housingoptions.org.uk/general_information/gi_publications_vpn_publications.html	Tried and failed to access this resource 30th May 2021
The Housing and Support Partnership (2011) lists: Housing Options: A Guide on Discretionary Trusts—available for families to download. www.housingoptions.org.uk/general_information/gi_publications_vpn_publications.html	Tried and failed to access this resource 30th May 2021
The Housing and Support Partnership (2011) lists: BILD: A guide on financial decision‐making. www.bild.org.uk/03books_pca.htm	Tried and failed to access this resource 30th May 2021
The Housing and Support Partnership (2011) lists: Housing Options: ‘Finding a Place to Live’—a guide to help with planning housing and support options—available to download free. www.housingoptions.org.uk/general_information/gi_publications_vpn_publications.html	Tried and failed to access this resource 30th May 2021
The Housing and Support Partnership (2011) lists: Dimensions UK & Housing Options: ‘My home and money’—a guide for people supporting adults with intellectual disabilities to manage their money, available to download free from Dimensions UK. www.housingoptions.org.uk/general_information/gi_publications_vpn_publications.html	Tried and failed to access this resource 30th May 2021
The Housing and Support Partnership (2011) lists: Growing Older with Learning Disabilities (GOLD)—a UK wide programme that operated throughout 1998–2002. It aimed to improve the lives of older people with intellectual disabilities. They set up a range of projects to increase our understanding of their concerns and how best they could be addressed. http://www.learningdisabilities.org.uk/our‐work/person‐centred‐support/gold	Tried and failed to access this resource 30th May 2021
The Housing and Support Partnership (2011) lists: Department of Health and Department for Communities and Local Government: Housing Resource Pack—designed to help people with intellectual disabilities have greater choice and control in where they live. Overall aim is to increase the number of adults with moderate to severe intellectual difficulties in settled accommodation. http://www.valuingpeoplenow.dh.gov.uk/valuing‐people‐now/housing	Tried and failed to access this resource 30th May 2021
The Housing and Support Partnership (2011) lists: Housing Options: HOLD programme—a factsheet downloadable for free. www.housingoptions.org.uk/general_information/gi_publications_vpn_leaflets.html	Tried and failed to access this resource 30th May 2021
The Housing and Support Partnership (2011) lists: Further information and advice about ‘Home Ownership for People with Long Term Disabilities’ is available from http://www.advanceuk.org and http://www.homesandcommunities.co.uk	Both sites accessed 30th May 2021
The Housing and Support Partnership (2011) lists: Housing Options: ‘Your Place to Live’—a guide to housing options for people with intellectual disabilities available to download free. www.housingoptions.org.uk/general_information/gi_publications_vpn_publications.html	Tried and failed to access this resource 30th May 2021
The Housing and Support Partnership (2011) lists: Housing Options: ‘Overview of Housing Choices’—a factsheet available free to download, which introduces a series of leaflets explaining how a disabled person may get housing along with whatever care or support they need. The leaflets are intended to help care managers, families, advocates and others who may play a role in a disabled person's life get a quick overview of the main housing possibilities. www.housingoptions.org.uk/general_information/gi_publications_vpn_leaflets.html	Tried and failed to access this resource 30th May 2021
The Housing and Support Partnership (2011) lists: Housing Options: ‘Residential Care Homes’—one of a set of leaflets available free to download from Housing Options [http://www.housingoptions.org.uk] explaining the residential care housing option. http://www.housingoptions.org.uk/general_information/gi_publications_vpn_leaflets.html	Tried and failed to access this resource 30th May 2021

## 4 DISCUSSION

We have identified a diverse range of evidence, which, collectively, suggests the same core message, namely, that transition‐related care and support of older people with intellectual disabilities and behaviours that challenge others remains lacking in crucial respects. The review has also highlighted a number of factors which appear to be critical in: (i) supporting this population as they transition from one context of care to another; and (ii) avoiding unnecessary transitions, notably at points of crisis, which are now discussed.

### 4.1 Implications of findings

In terms of supporting effective transition, we identified a number of systems issues that need to be addressed. The importance of proactive preparation and planning across multiple areas of activity is clear. These areas include: professional involvement in supporting transition planning amongst people with intellectual disabilities and their family carers (Forrester‐Jones, 2019; Slevin et al., 2011); preparing people with intellectual disabilities for independence by supporting them to develop appropriate skills/confidence (Slevin et al., 2011); multi‐disciplinary and multi‐agency collaboration to achieve the required holistic planning and delivery of support (Bissell et al., 2005; Hubert & Hollins, 2010; Leaning & Adderley, 2015; The Housing Support Partnership, 2011) and, the strategic commissioning of suitable community based accommodation (Perry et al., 2013; Slevin et al., 2011; The Housing Support Partnership, 2011). The critical need for a ‘whole family’ approach to transition planning is highlighted (Forrester‐Jones, 2019; Slevin et al., 2011), as is the involvement of social workers in supporting decision‐making for both older people with intellectual disabilities and behaviours that challenge others as they age and their families (Forrester‐Jones, 2019).

In terms of avoiding unwanted/crisis transition, the review has identified a need for sustained, proactive professional involvement to underpin responsive care. Again, the need for full multi‐disciplinary and multi‐agency input, including that from specialist community teams as appropriate (Slevin et al., 2011), as a basic and indisputable component of care and support was consistently demonstrated. This includes in respect of needs assessments enshrined in The Care Act 2014 (England), and the subsequent development of relevant packages of support (Forrester‐Jones, 2019; Slevin et al., 2011). Timely engagement with health services will help to identify the onset of conditions that may lead to or exacerbate existing behaviours that challenge others (Bissell et al., 2005; Forrester‐Jones, 2019; Slevin et al., 2011). The effectiveness of all such care, whether delivered by professional or lay carers, is premised on training and support of family carers and staff working in both community and intellectual disability specific settings (Perry et al., 2011; Slevin et al., 2011) to understand and respond appropriately. Given the particular importance of dementia in older age, training should be provided to family and front‐line professional carers in relation to both the recognition of the early symptoms, as well as the care of people with intellectual disabilities who develop dementia (Slevin et al., 2011).

More fundamentally, in terms of the principles underpinning these systems of care, evidence prioritises the need to privilege the social identity and connectedness of people with intellectual disabilities and behaviours that challenge others through, for example: person‐centred support to enable them to live independent lives; honouring their relationships (for example, through locally based placements that allow people to maintain their family and social links); and, firmly embedding people in local communities (e.g., by the otherwise taken‐for‐granted step of providing a home in these communities). These principles foreground the *values* underpinning care. It was the particular attitude and supportive approach of a community residential manager that Hubert and Hollins (2010) associated with improved quality of life of people on transition to community residential living. It was also the compassion, commitment and perseverance of the clinical psychology lead that resulted in the successful transition of the older man with behaviours that challenge others (Leaning & Adderley, 2015).

These examples directly reference our opening comments regarding the critical need for ‘behaviours that challenge others’ to be understood as a socially constructed phenomenon, predicated on the understanding of *all* involved within particular inter‐personal, organisational and systems contexts of interaction. As our analysis has demonstrated, it is these social dynamics that are critical to promoting and impeding effective transitioning. In this context, although the need for appropriate training and support of professional staff is indubitable, equally the care delivered by those staff, and the organisations and systems within which they work, must be predicated on values that promote the human rights of those for whom they care (see, e.g., Banks et al., 2021; Farrell et al., 2010; Hastings et al., 2018; Olivier‐Pijpers et al., 2019; Osgood, 2019; Willems et al., 2014). Our review demonstrates a clear link between such values, effective working within and across different environments and systems of care, and successful transition.

It is also clear from the literature currently available that the carers of people with intellectual disabilities and behaviours that challenge others are often central to successful transition from one environment of care to another (Brennan et al., 2020; Carers Trust, 2020; Lee & Burke, 2020). The reasons include: their willingness and ability to engage in transition planning; their own support needs and the extent to which these are met; and, their central role in considering options for alternative and suitable care. A lack of locally relevant resources in the form of, for example, written guides, to help decision‐making of older people with intellectual disabilities and behaviours that challenge others, their families and care professionals is an important gap too, particularly given the increase in life expectancy for this group.

### 4.2 Study strengths and limitations

In terms of study limitations, as a rapid scoping review, the possibility that we have missed some relevant evidence remains. That we made strenuous efforts to read a wide body of potentially relevant evidence, the vast majority of which we eventually excluded, reduces this possibility. In terms of strengths, we adopted an enhanced rapid review process, including the use of multiple reviewers at all stages.

Focusing our search strategy on ‘challenging behaviour’ (and related synonyms and terminology variations) as opposed to specific conditions that may be associated with behaviours that challenge others (e.g., dementia; mental and/or physical ill health) and/or specific forms of behaviour (e.g. self‐injury; violence etc.) runs the risk of having missed articles that pertained to our population of interest. However, a trial of that alternative approach returned a vast number of returns that would have been unmanageable for the purposes of a rapid scoping review. Moreover, an initial scan of the returns showed no relevance to our research question. Therefore, we made a pragmatic decision to build a search strategy around the key terms of ‘intellectual disability’, ‘challenging behaviour’ and ‘ageing’.

Our included evidence encompassed male and female participants, assessed across mild to severe/profound intellectual disability, and with a range of physical and mental health conditions. Autism was most frequently cited (*n* = 5 studies), followed by mental ill‐health (*n* = 4 studies), epilepsy (*n* = 2 studies), genetic conditions (*n* = 1 study) and cerebral palsy (*n* = 1 study). As ‘mental ill‐health’ was not further defined, we are unable to determine its particular presentation. Therefore, we can say that our findings are broadly relevant to the population of older people with intellectual disabilities and behaviours that challenge others, including in respect of gender, degree of intellectual disability and health status generally. Such breadth means that our findings may have less relevance to a particular population of people with intellectual disability, for example, those not addressed by our included evidence (e.g., people with dementia) and/or those whose degree of intellectual disability and/or attendant health status may prompt a need for specific care and support.

We intentionally did not adhere to a specific definition of ‘behaviours that challenge others’. This enabled us to investigate how the term was understood and applied in the literature. Only one of the included sources of evidence (Slevin et al., 2011) provided a working definition, using the oft‐cited Emerson reference (1995). The authors also sought to contextualise their approach, prioritising a need to understand ‘challenging behaviour’ as inherently relational. The remaining sources of evidence either did not provide any indication of the type of behaviour or simply gave examples (including those set out in the Aberrant Behaviour Checklist (Aman & Singh, 1986)). In neither case was any discussion offered of the context in which this behaviour was being understood. Based on our included evidence, we suggest that our themes and recommendations relate to a broad range of behaviours that challenge others, as these are socially located.

We found very little evidence specifically focused on older people with intellectual disabilities and behaviours that challenge others in the context of transitioning to different contexts of care. We were therefore largely dependent on evidence that addressed our review question as part of a broader focus, typically either older people with intellectual disabilities or people with intellectual disabilities and behaviours that challenge others. From our included evidence, we extracted relevant content. This required close reading of many items of evidence that ended up including no relevant content whatsoever. Our review is therefore based on a limited body of relevant evidence, reflecting a current dearth of research focusing on our particular population of interest. We are also mindful that a majority of evidence included in this review was over 10 years old at the time of writing, pre‐dating a number of significant developments in the UK context, notably the impact of austerity (Malli et al., 2018), the roll out of annual health checks by primary care (PHE, 2016b) and the *Transforming Care* programme (NHS England et al., 2015). Although we have been able to deliver some key learning concerning current planning and implementation of transition‐related care and support for older people with intellectual disabilities and behaviours that challenge others, it is clear that gaps in research remain.

### 4.3 Implications for policy and practice

Our review highlights that the needs of older people with intellectual disabilities and behaviours that challenge others must be afforded greater priority within health and social care policy and commissioning practices. Wider research exploring intellectual disability and ageing (although not focused on the experiences of people with behaviours that challenge others) has highlighted the need for social care staff training to facilitate more nunaced and proactive approaches to support people as they grow older (Alftberg et al., 2021; Northway et al., 2017). The promotion of healthy ageing amongst people with intellectual disabilities relies on services and frontline staff understanding the specific health needs of this population, clearly evidenced through large‐scale longitudinal research (de Leeuw et al., 2022; McCausland et al., 2016). The policy challenge lies in disseminating this knowledge effectively to practitioners, family carers and people with intellectual disabilities, while developing approaches to healthy ageing in services that are person‐centred, nuanced and responsive. Involving people with intellectual disabilities and their families/advocates (where possible) is also critical to ensuring that well‐planned decisions are made—decisions that take into account people's whole lives, including their relationships, homes, activities and hobbies, as well as their health needs.

Research has shown that despite policy commitments to ageing in place, progress in developing or adapting existing accommodation for people with intellectual disabilities as they age has been slow across a range of international contexts (Bigby, 2010). As this review has highlighted, commissioning and delivering suitable accommodation for people with intellectual disabilities and behaviours that challenge other is critical. It underpins family carers' ability to trust that suitable alternatives for their adult child exist beyond the family home, and it is necessary to avoid unsuitable placements in the event that a person's health deteriorates quickly (Bigby et al., 2011). Fundamentally, contemporary policy for people with intellectual disabilities has not been developed to take account of people growing older. As a consequence, systems are not designed to support comprehensive commissioning for this population, and in many cases prohibit effective, timely and inclusive discussions of future care plans (Brennan et al., 2020), with consequent distressing, avoidable, and costly crisis management. This needs to change.

### 4.4 Implications for research

The themes identified also highlight a range of issues that require further research to improve the support of this population. One particularly striking finding was the absence of empirical research documenting the health and social care services that this group access as they age. From this review, it is not clear to what extent this population are enabled to age ‘in place’ (Perry et al., 2011). In addition, we were not able to decipher the settings that people transition to following a significant change in their health status, a family crisis, or as they reach end of life. This suggests to us that research is urgently needed to assess the types of services that this population can and do access as they age, the quality of those services, and the extent to which local commissioners are planning ahead for this population.

## FUNDING INFORMATION

This review is part of the Growing Older Planning Ahead research project, funded by the National Institute for Health and Care Research (NIHR) under its Health Services and Delivery Research Programme (NIHR129491). The views expressed are those of the authors, and not necessarily those of the NHS, the NIHR or the Department of Health and Social Care.

## CONFLICT OF INTEREST

The authors declare no conflicts of interest.

## Supporting information


**Appendix 1:** Search Strategies.
**Appendix 2**: Study inclusion screening form.
**Appendix 3**: Data extraction form.

## Data Availability

The data that supports the findings of this review are available from the corresponding author on request.

## Footnotes

^1^
The Growing Older Planning Ahead Study: Improving the support for older people with learning disabilities and behaviours that challenge, family and professional carers and supporting end of life care planning for carers. Funded by the National Institute for Health and Care Research (NIHR129491).

^2^
We adopt a wide‐ranging definition of ‘care contexts’ for the purpose of this review, which encompasses the following environmental factors: service type (i.e., is the person living in a congregate setting, independently, or in a family home); provider type (NHS/local authority, private provider, third sector organisation, family care); relationships (who provides the care, for example, different family members; paid carers; personal assistants); place (the geographical location of care, taking particular note of the urban/rural dimension); and commissioning and funding arrangements. Our definition of care contexts also takes into account the dynamic and fluid ways in which different contextual factors interact at the micro, meso and macro levels. Our decision to focus on both transitions out of the family home as well as transitions *between* different service contexts was informed by findings emerging from a concurrent work package on the project and discussions with our advisory group. Both indicated that there was a population of older people with intellectual disabilities and labels of behaviours that challenge others identified as needing to transition from one form of care (e.g., supported living, residential care) into another (e.g., nursing care) due to age related health and care needs.

^3^
Our rationale for defining ‘older adults with intellectual disabilities and behaviours that challenge others’ as 40+ in this context was twofold, and reflects the definition used in the wider research project. 40+ for people with intellectual disabilities has been selected to reflect the early onset of some chronic health conditions, such as dementia, for this group (Ballard et al., 2016). In addition, this age range enables us to capture the perspectives of more family carers who might be described as ‘older’ and in need of new/additional support—that is, family carers in their early 60s+.
