# Supplementary material for: Transitions for older people with intellectual disabilities and behaviours that challenge others: A rapid scoping review
Source: J Appl Res Intellect Disabil. 2022 Nov 26;36(2):207–29. doi: 10.1111/jar.13054 (PMC10098666; doi:10.1111/jar.13054)
Supplement: Supplementary file 1 — Appendix 1: Search Strategies. Appendix 2: Study inclusion screening form. Appendix 3: Data extraction form. [file JAR-36-207-s001.docx]

**Appendix One: Search Strategies**

| Database | Search strategy |
| --- | --- |
| CINAHL | ((("challenging behavior" OR "challenging behaviour" OR "problem behavior" OR "problem behaviour" OR aggression OR aggressive OR "behaviour of concern" OR "behavior of concern" OR "risky behaviour" OR "risky behavior" OR "high risk behaviour" OR "high risk behavior" OR "self-injury" OR "self-harm*" OR "antisocial behaviour" OR "antisocial behavior" OR "anti social behavior" OR "anti social behaviour" OR "unacceptable behaviour" OR "unacceptable behavior" OR "inappropriate behaviour" OR "inappropriate behavior" OR "worrying behaviour" OR "worrying behavior") ADJ5 ("developmental delay" OR "developmental disability" OR "developmental disabilities" OR "learning disability" OR "learning disabilities" OR "intellectual disability" OR "intellectual disabilities" OR "intellectual disorder" OR "intellectual disorders" OR "intellectual impairment" OR "intellectual impairments" OR "intellectual handicap" OR "learning difficulty" OR "learning difficulties" OR "cognitive disability" OR "cognitive disabilities" OR "cognitive impairment" OR "cognitive impairments" OR "mental retardation" OR "mental handicap")) ADJ5 (oldest OR aging OR ageing OR elders OR elderly OR senior OR seniors OR "middle aged" OR frail OR frailty OR midlife OR "mid life" OR "middle life" OR "late life" OR "later life" OR "mature adults" OR "end of life" OR geriatric)).ti,ab |
| Google | Customised data range 2001-2020, pages 1-5 searched  “learning disabilities behaviour that challenge oldest old people united kingdom pdf”  “oldest old people learning disabilities behaviour that challenges united kingdom pdf”  “intellectual disability challenging behaviour elderly united kingdom pdf”  “elderly intellectual disability behaviour that challenges united kingdom pdf” |
| Google Scholar | Customised data range 2001-2020, pages 1-5 searched  “older people learning disabilities behaviour that challenges united kingdom”  “older people intellectual disabilities aggression united kingdom”  “seniors learning difficulties self harm united kingdom”  “intellectual disabilities behaviour that challenges oldest old people united kingdom”  “learning disabilities behaviour that challenge oldest old people united kingdom” |
| HMIC | ((("challenging behavior" OR "challenging behaviour" OR "problem behavior" OR "problem behaviour" OR aggression OR aggressive OR "behaviour of concern" OR "behavior of concern" OR "risky behaviour" OR "risky behavior" OR "high risk behaviour" OR "high risk behavior" OR "self-injury" OR "self-harm*" OR "antisocial behaviour" OR "antisocial behavior" OR "anti social behavior" OR "anti social behaviour" OR "unacceptable behaviour" OR "unacceptable behavior" OR "inappropriate behaviour" OR "inappropriate behavior" OR "worrying behaviour" OR "worrying behavior") ADJ5 ("developmental delay" OR "developmental disability" OR "developmental disabilities" OR "learning disability" OR "learning disabilities" OR "intellectual disability" OR "intellectual disabilities" OR "intellectual disorder" OR "intellectual disorders" OR "intellectual impairment" OR "intellectual impairments" OR "intellectual handicap" OR "learning difficulty" OR "learning difficulties" OR "cognitive disability" OR "cognitive disabilities" OR "cognitive impairment" OR "cognitive impairments" OR "mental retardation" OR "mental handicap")) ADJ5 (oldest OR aging OR ageing OR elders OR elderly OR senior OR seniors OR "middle aged" OR frail OR frailty OR midlife OR "mid life" OR "middle life" OR "late life" OR "later life" OR "mature adults" OR "end of life" OR geriatric)).ti,ab |
| NHS Evidence | – first 50 results scanned / Primary research  “older people learning disabilities behaviour that challenges”  “older people intellectual disabilities aggression”  “seniors learning difficulties self harm”  “challenging behaviour older adults with learning disability”  NHS Evidence/ Secondary evidence – first 50 results scanned  “challenging behaviour older adults with learning disability”  “seniors learning difficulties self harm”  “oldest old people learning disabilities behaviour that challenges” |
| Scopus | TITLE-ABS(( ( "challeng* behav*" ) OR ( behav* W/5 challeng* ) OR ( "problem* behav*" ) OR ( aggression ) OR ( aggressive W/5 behav*) OR ( "behav* of concern" ) OR ( risky W/5 behav* ) OR ( "high risk" W/5 behav* ) OR ( "self-injur*" ) OR ( "self-harm*" ) OR ( antisocial W/5 behav* ) OR ( anti-social W/5 behav* ) OR ( unacceptable W/5 behav* ) OR ( inappropriate W/5 behav* ) OR ( worrying W/5 behav* ) ) AND ( ( ( "developmental* disab*" ) OR ( "developmental delay" ) OR ( "learning disab*" ) OR ( "intellect* disab*" ) OR ( "intellect* disorder*" ) OR ( "intellect* impair*" ) OR ( "intellect* handicap*" ) OR ( "learning difficult*" ) OR ( "cognitive disab*" ) OR ( "cognitive impair*" ) OR ( "mental retard*" ) OR ( "mental handicap*" ) ) AND ( ( "older adult*" ) OR ( aging ) OR ( ageing ) OR ( elderly ) OR ( senior* ) OR ( "adult child*" ) OR ( "old age" ) OR ( "middle age*" ) OR ( frail* ) OR ( midlife ) OR ( "mid-life" ) OR ( "middle life" ) OR ( "late* life" ) OR ( "aged adult*" ) OR ( "mature adult*" ) ) ) ) Limit to SCOPUS's UK geographical filter |
| TRIP | ("challenging behavior" OR "challenging behaviour" OR "problem behavior" OR "problem behaviour" OR aggression OR aggressive OR "behaviour of concern" OR "behavior of concern" OR "risky behaviour" OR "risky behavior" OR "high risk behaviour" OR "high risk behavior" OR "self-injury" OR "self-harm*" OR "antisocial behaviour" OR "antisocial behavior" OR "anti social behavior" OR "anti social behaviour" OR "unacceptable behaviour" OR "unacceptable behavior" OR "inappropriate behaviour" OR "inappropriate behavior" OR "worrying behaviour" OR "worrying behavior") AND ("developmental delay" OR "developmental disability" OR "developmental disabilities" OR "learning disability" OR "learning disabilities" OR "intellectual disability" OR "intellectual disabilities" OR "intellectual disorder" OR "intellectual disorders" OR "intellectual impairment" OR "intellectual impairments" OR "intellectual handicap" OR "learning difficulty" OR "learning difficulties" OR "cognitive disability" OR "cognitive disabilities" OR "cognitive impairment" OR "cognitive impairments" OR "mental retardation" OR "mental handicap") AND (geriatric or oldest OR aging OR ageing OR elders OR elderly OR senior OR seniors OR "middle aged" OR frail OR frailty OR midlife OR "mid life" OR "middle life" OR "late life" OR "later life" OR "mature adults" OR "end of life" or geriatric))- UK guidelines |
| WoS | TS=(("challenging behavior" OR "challenging behaviour" OR "problem behavior" OR "problem behaviour" OR aggression OR aggressive OR "behaviour of concern" OR "behavior of concern" OR "risky behaviour" OR "risky behavior" OR "high risk behaviour" OR "high risk behavior" OR "self-injury" OR "self-harm*" OR "antisocial behaviour" OR "antisocial behavior" OR "anti social behavior" OR "anti social behaviour" OR "unacceptable behaviour" OR "unacceptable behavior" OR "inappropriate behaviour" OR "inappropriate behavior" OR "worrying behaviour" OR "worrying behavior") AND ("developmental delay" OR "developmental disability" OR "developmental disabilities" OR "learning disability" OR "learning disabilities" OR "intellectual disability" OR "intellectual disabilities" OR "intellectual disorder" OR "intellectual disorders" OR "intellectual impairment" OR "intellectual impairments" OR "intellectual handicap" OR "learning difficulty" OR "learning difficulties" OR "cognitive disability" OR "cognitive disabilities" OR "cognitive impairment" OR "cognitive impairments" OR "mental retardation" OR "mental handicap") AND (geriatric or oldest OR aging OR ageing OR elders OR elderly OR senior OR seniors OR "middle aged" OR frail OR frailty OR midlife OR "mid life" OR "middle life" OR "late life" OR "later life" OR "mature adults" OR "end of life" or geriatric) ) - limit to Web of Science's filter for UK |

**Appendix 2: Study inclusion screening form**

| What are the health and social care needs, experiences, service interventions and resources of and for older people with intellectual disabilities and behaviours that challenge others as they move to different care contexts in the UK? | |
| --- | --- |
| Study ID (family name of first author & year of publication + letter if more than one per year e.g. Smith 2019a) |  |
| Form completed by |  |
| Date of completion |  |
| Are there any other records of the same study? Yes/No/Unclear |  |
| Criterion | Yes/No/Unclear, with explanation if required |
| 1. Be published in English |  |
| 2. Be published / made available since 2001 |  |
| 3. Concern older (40+) adults with intellectual disabilities and behaviours that challenge others resident in the UK |  |
| 4. Concern older adults with intellectual disabilities and behaviours that challenge others who are moving to different care contexts |  |
| ***All of the answers to 1., 2., 3. and 4. must be Yes to Include.*** |  |
| 5. Report empirical research focused on health and social care needs and experiences |  |
| 6. Report a systematic review of empirical research focused on health and social care needs and experiences |  |
| 7. Report service interventions targeting health and social care needs |  |
| 8. Report resources relevant to health and social care needs. |  |
| ***At least one of the answers to 5., 6., 7. and 8. must be Yes to Include*** |  |
| **Decision - Include / Exclude / More information needed before decision can be made**  **(*please specify information required*)** |  |

**Appendix 3: Data extraction form**

| **Background Information** | |
| --- | --- |
| **Date of data extraction & by whom** |  |
| **Study ID** | **Title** |
|  | **Author(s)** *(e.g. surname, initials and affiliation)* |
|  | **Citation** *(journal, year, vol, pg.)* |
|  | **Unpublished source** *(from where? e.g. for a MSc/PhD, give the name of the university)* |
| **Evidence Characteristics** | |
| **Type** *(e.g. journal article, service evaluation report, patient/client resource (e.g. guide/advice), policy document)* |  |
| **Aims & Objectives / Purpose** |  |
| **How assembled** *(e.g. via research, other type of investigation, parliamentary committee, expert review etc.)* |  |
| **PPI involvement** *(i.e. presence / nature of co-production / co-research)* |  |
| **Geographical area of / to which evidence applies** *(e.g. where study conducted / jurisdiction of parliamentary committee etc.)*  *If possible/relevant, please state if evidence clarifies the context as urban or rural* |  |
| **Target group / population** *(this can include any type of ‘case’ – patients, health care professionals, services etc.)* |  |
| **From where were cases recruited / sourced? How were they accessed?** |  |
| **If the evidence concerns services/resources – how are they funded?** *(specifically, any reference to personal (health) budgets)* |  |
| **Details of methods by which evidence assembled** *(e.g. methods of data collection, did it involved research/ other participants e.g. members of a committee / expert review group / charity), study intervention, comparator and outcomes etc.)* |  |
| **Details of methods of analysis** *(e.g. quantitative, qualitative or mixed methods / consensus techniques / expert derived)* |  |
| **Results / Findings** | |
| **Number and characteristics of included cases** *(e.g. study sample, experts etc.)* |  |
| **Substantive results / findings**  *If relevant, please state if analysis addresses if/how the issue of rural Vs urban provision impacts on models of services and support*  *If relevant, please state if analysis addresses the issue of protected characteristics- ethnicity, gender, sexuality*  *If relevant, please state if analysis addresses the issue of how the service/provision is funded (i.e. social care; health; personal budget etc.)* |  |
| **Authors’ stated conclusions** |  |
| **Authors’ stated implications of results / findings for policy, practice and/or research** |  |
| **Authors’ stated limitations** |  |
